# Preventing relapse in ulcerative colitis remission maintenance: machine learning prediction and reinforcement-learning herbal prescription optimization with external validation

**DOI:** 10.3389/fmed.2026.1911189

**Published:** 2026-08-24

**Authors:** Yan Li, Jie Liu, Jian Kang

**Affiliations:** 1 The Affiliated Yongchuan Hospital of Chongqing Medical University, Chongqing, China; 2 People’s Hospital of Shapingba District, Chongqing, China; 3 Hospital of Chengdu University of Traditional Chinese Medicine, Chengdu, Sichuan, China

**Keywords:** calprotectin, machine learning, personalized treatment, reinforcement learning, remission maintenance, traditional Chinese medicine, ulcerative colitis

## Abstract

**Introduction:**

Maintaining remission in ulcerative colitis (UC) with traditional Chinese herbal medicine is challenging because fixed-intensity prescriptions may not accommodate changes in inflammatory burden. This study aimed to predict 52-week relapse and develop a data-driven policy for sequential adjustment of herbal treatment intensity.

**Methods:**

We analysed a development cohort of 1,181 patients receiving herbal maintenance therapy and an independent external validation cohort of 96 patients. Twenty-one baseline clinical, biomarker, herbal-dose and behavioral variables, expanded to 31 features after one-hot encoding, were used to train and compare seven machine-learning models through stratified five-fold cross-validation and external validation. Discrimination, calibration and decision-curve net benefit were evaluated. Remission maintenance was subsequently modelled as a sequential decision problem using 4,141 scheduled-visit transitions. A gradient-boosted reward model was used for model-based off-policy evaluation of a net-clinical-benefit policy that penalized unnecessary treatment intensification in clinically quiescent patients.

**Results:**

Calibrated logistic regression provided the best overall performance, with an internal cross-validation area under the receiver operating characteristic curve (AUROC) of 0.783 (95% confidence interval [CI], 0.755–0.809) and an external AUROC of 0.710 (95% CI, 0.600–0.820). The corresponding Brier scores were 0.180 (95% CI, 0.169–0.191) and 0.215 (95% CI, 0.170–0.263), respectively. Medication adherence was the strongest predictor of relapse, followed by fecal calprotectin, C-reactive protein and Coptidis Rhizoma dose. The learned policy increased herbal intensity with increasing fecal calprotectin and achieved a higher estimated mean reward than observed clinician behavior (0.552 ± 0.018 versus 0.063 ± 0.021). Policy-concordant visits were associated with a higher next-visit remission rate than discordant visits (70.0% versus 59.6%).

**Discussion:**

Combining interpretable relapse prediction with reinforcement learning offers a personalized decision-support framework for optimizing herbal treatment intensity during UC remission maintenance. Because the policy was derived and evaluated using observational data and model-based off-policy methods, prospective clinical evaluation is required before routine implementation.

## 1 Introduction

Ulcerative colitis (UC) is a chronic, relapsing–remitting inflammatory bowel disease whose long-term management hinges less on inducing remission than on keeping patients in remission once it is achieved (1, 2). Even when mucosal healing is attained, a substantial fraction of patients relapse within a year, and each relapse carries cumulative risks of corticosteroid exposure, hospitalization, colectomy and diminished quality of life (3). The maintenance phase is therefore the dominant determinant of the lifetime burden of the disease, yet it is also the phase in which therapeutic decisions are most weakly evidence-guided, because trials are concentrated on the induction window and because maintenance regimens are frequently held fixed for long periods regardless of subclinical disease activity.

In many parts of East Asia, and increasingly worldwide, herbal therapy rooted in traditional Chinese medicine (TCM) is used alongside mesalazine to sustain remission in UC. Decoctions built around herbs such as Coptidis Rhizoma, Indigo naturalis, Sanguisorba and Pulsatilla are believed to exert anti-inflammatory and mucosal-protective effects (4, 5). Network-pharmacology studies have begun to rationalize these effects at a molecular level (6, 7). However, herbal maintenance prescriptions in routine practice are usually prescribed at a fixed intensity, titrated mostly by symptoms rather than by objective markers of inflammation. This creates two complementary failure modes: under-treatment of patients with rising subclinical inflammation who go on to relapse, and over-treatment of quiescent patients who are exposed to unnecessary polypharmacy, cost and the safety-monitoring burden that agents such as Indigo naturalis demand.

A natural way to reframe remission maintenance is as a sequential decision problem. At each scheduled visit, a clinician observes the patient’s current inflammatory state, chooses how intensively to dose the herbal regimen, and later observes the consequences for disease activity. Supervised machine learning has shown promise for predicting inflammatory-bowel-disease outcomes from routinely collected data (8, 9), and reinforcement learning (RL) has been explored for optimizing treatment in sepsis, anaesthesia and chronic disease (10, 11). The two approaches are complementary: an accurate, interpretable risk model identifies who is likely to relapse and why, while an RL policy translates that risk into a concrete, individualized dosing recommendation. To our knowledge, these tools have not been jointly applied to personalize herbal-intensity decisions for UC remission maintenance.

The gap we address is therefore both methodological and clinical. Clinically, fixed herbal regimens ignore the substantial patient-to-patient and visit-to-visit heterogeneity in inflammatory trajectory. Methodologically, prior decision-support work for UC has emphasized one-shot relapse prediction rather than the sequential treatment-adjustment problem that maintenance actually poses, and has rarely been validated on an independent cohort or evaluated for calibration and net clinical benefit rather than discrimination alone.

Recent advances in clinical decision-support systems for ulcerative colitis have primarily focused on optimizing conventional medical therapies, particularly mesalazine, corticosteroids, immunomodulators, and biologic agents. Machine-learning approaches have been applied to predict clinical relapse, corticosteroid dependence, mucosal healing, and response to biologic therapy using clinical, laboratory, endoscopic, and multi-omics data. However, these studies generally provide static risk prediction only and do not generate individualized treatment recommendations. Moreover, existing artificial intelligence-based decision-support systems have almost exclusively been developed for conventional pharmacological therapies, whereas personalized optimization of traditional Chinese herbal medicine has received little attention despite its widespread use for long-term remission maintenance in East Asia. To our knowledge, no previous study has integrated relapse prediction with reinforcement learning to dynamically optimize sequential herbal treatment intensity according to an individual patient’s evolving relapse risk.

The primary objective of this study was to improve individualized remission maintenance in patients with ulcerative colitis receiving traditional Chinese herbal medicine by developing a clinically applicable decision-support framework. Specifically, we first developed and externally validated machine-learning models for predicting the risk of 52-week clinical relapse, and subsequently applied reinforcement learning to optimize sequential herbal treatment intensity according to patients’ evolving relapse risk. Rather than proposing new machine-learning algorithms, this study aimed to translate artificial intelligence techniques into clinically meaningful tools for personalized relapse prevention and treatment decision-making.

## 2 Related work and background

### 2.1 Machine learning for inflammatory-bowel-disease outcomes

Machine learning has been increasingly applied to predict inflammatory bowel disease (IBD) outcomes, including relapse, response to biologics, hospitalization and the need for surgery, using electronic-health-record data, laboratory biomarkers and patient-reported measures (8, 9, 12). Tree ensembles such as random forests and gradient boosting are popular because they accommodate mixed data types and nonlinear interactions, whereas penalized logistic regression remains attractive for its transparency and stable calibration in small cohorts (13–15). A recurring theme in this literature is that discrimination measured by the area under the receiver-operating-characteristic curve (AUROC) is often reported without accompanying calibration or external validation, which limits clinical trust and reproducibility (16, 17). Our work follows recent recommendations by reporting calibration, decision-curve net benefit and external validation alongside discrimination.

### 2.2 Reinforcement learning for treatment optimization

Reinforcement learning frames treatment as a sequence of state-dependent actions chosen to maximize a cumulative reward, and has been investigated for dynamic treatment regimes in critical care, anaesthetic dosing, diabetes management and oncology (10, 18, 19). Because randomized exploration is rarely ethical in patient care, most medical RL relies on off-policy evaluation from observational data, in which the value of a proposed policy is estimated without deploying it (11, 20). Model-based off-policy evaluation, in which a learned reward or transition model substitutes for missing counterfactuals, is a pragmatic choice when trajectories are short and the action space is small, but it inherits the assumptions of the underlying model and is sensitive to distributional shift (21). We adopt a deliberately constrained, low-dimensional formulation that keeps these assumptions inspectable.

### 2.3 Herbal therapy and network pharmacology for ulcerative colitis

Traditional Chinese herbal therapy for UC is typically organized around syndrome differentiation, in which patients are assigned a TCM pattern (for example Damp-Heat or Spleen-Deficiency) that guides herb selection (4, 22). Several core herbs used in maintenance prescriptions, including Coptidis Rhizoma and Indigo naturalis, have documented anti-inflammatory activity, and network-pharmacology analyses have proposed multi-target mechanisms involving cytokine signalling and mucosal barrier function (5, 7, 23). Indigo naturalis in particular has shown efficacy signals in UC but requires safety monitoring, reinforcing the clinical value of avoiding unnecessary intensification (24). Despite this growing mechanistic understanding, the question of how to titrate herbal intensity over time for an individual patient has remained largely empirical, which is precisely the gap a data-driven sequential policy can help to fill.

## 3 Materials and methods

### 3.1 Study cohorts

We analysed two independent cohorts of patients with ulcerative colitis (UC) who were in clinical remission and receiving maintenance therapy with traditional Chinese herbal medicine. The development cohort consisted of 1,200 consecutive patients enrolled at the participating center between January 2018 and December 2023. After excluding 19 patients who were lost to follow-up or lacked a documented relapse outcome, 1,181 patients were included in the final analysis. The primary outcome was clinical relapse within 52 weeks after enrolment (relapse_52wk). Clinical relapse was defined as recurrence of active UC requiring treatment escalation, accompanied by a partial Mayo score ≥3 with an increase of at least 2 points compared with the remission visit, together with at least one objective indicator of inflammatory activity, including fecal calprotectin >250 μg/g, C-reactive protein >5 mg/L, or endoscopic evidence of active mucosal inflammation when available. Patients who required systemic corticosteroids, initiation of biologic therapy, hospitalization for UC, or colectomy during follow-up were also classified as having experienced clinical relapse. This composite outcome definition was established before data analysis and was applied consistently in both the development and external validation cohorts. The external validation cohort consisted of 100 consecutive patients prospectively enrolled at the Hospital of Chengdu University of Traditional Chinese Medicine between January 2024 and December 2024. Of these, 96 patients completed the 52-week follow-up and had complete outcome data for external validation. This cohort was approved by the institutional ethics committee (Approval No. 2024KL-087). The external cohort was geographically and temporally independent of the development cohort and was not involved in any stage of model development or tuning, thereby providing a rigorous assessment of model transportability in accordance with the Transparent Reporting of a Multivariable Prediction Model for Individual Prognosis or Diagnosis (TRIPOD) recommendations (25, 26). Baseline characteristics were formally compared between the two cohorts to characterize case-mix differences: continuous variables were compared with the Mann–Whitney U test (robust to the skew of biomarkers such as fecal calprotectin) and categorical variables with Pearson’s chi-square test (Fisher’s exact test where any expected cell count was <5), with two-sided *p* < 0.05 considered significant; because these comparisons are descriptive, no adjustment for multiple testing was applied. The development cohort had an observed 52-week relapse rate of 36.5%, and the external Chengdu cohort had a relapse rate of 43.8%, reflecting a modestly higher-risk referral population at the external site.

Patients were eligible for inclusion if they met all of the following criteria: (1) age ≥18 years; (2) a confirmed diagnosis of ulcerative colitis based on established clinical, endoscopic, histological, and radiological criteria; (3) achievement of clinical remission before enrollment, defined as a partial Mayo score ≤2 with no individual subscore >1; (4) receipt of maintenance therapy with traditional Chinese herbal medicine throughout the remission period; and (5) availability of complete baseline clinical data and planned 52-week follow-up.

Patients were excluded if they (1) had Crohn’s disease, indeterminate colitis, or other non-UC gastrointestinal inflammatory disorders; (2) underwent colectomy before completion of follow-up; (3) had concurrent malignant tumors, severe hepatic, renal, or cardiovascular diseases, or other serious systemic illnesses that could influence disease assessment; (4) were pregnant or breastfeeding; (5) discontinued herbal maintenance therapy during follow-up for reasons unrelated to disease progression; or (6) had incomplete baseline information or missing primary outcome data.

No formal *a priori* sample size calculation was performed because this study included all consecutive eligible patients available during the prespecified study periods. The development cohort size was therefore determined by the number of patients meeting the eligibility criteria and having complete 52-week outcome data. After exclusion of patients with incomplete follow-up, 1,181 patients were included, of whom 431 experienced clinical relapse. Following one-hot encoding, 31 candidate features were entered into model development, yielding an events-per-variable (EPV) ratio calculated as

$$\mathrm{EPV}=\frac{N_{\text{events}}}{N_{\text{predictors}}}=\frac{431}{31}=13.9$$

Which exceeds the commonly recommended minimum of 10 events per predictor variable for prediction model development. The external validation cohort similarly included all consecutive eligible patients prospectively enrolled during the predefined study period. Of the 100 enrolled patients, 96 had complete follow-up data (42 relapse events and 54 non-relapse events) and were therefore included in the external validation. No separate formal sample size calculation was performed for the external validation cohort; its size was determined by the number of eligible patients available at the independent validation centre.

### 3.2 Predictors and preprocessing

Each patient was described by 21 baseline variables: 17 numeric and 4 categorical. The numeric variables were age, body-mass index, disease duration, baseline partial Mayo score (27), fecal calprotectin (28), C-reactive protein, erythrocyte sedimentation rate, haemoglobin, albumin, platelet count, maintenance mesalazine dose (29), and the daily doses of four core herbs (Coptidis Rhizoma, Indigo naturalis, Sanguisorba and Pulsatilla), together with medication adherence (30, 31) and travel distance to the treating clinic. The categorical variables were sex, Montreal disease extent (E1/E2/E3) (32), smoking status, and the differentiated TCM syndrome (one of Damp-Heat, Spleen-Deficiency, Spleen-Kidney-Yang, Liver-Spleen, Blood-Stasis or Cold-Heat-Complex). Categorical variables were one-hot encoded, yielding 31 model features. The small fraction of missing values was imputed with the median (numeric) or mode (categorical) computed on the training data only, and rows with a missing relapse outcome were excluded rather than imputed. Numeric features were standardized within each cross-validation training fold to prevent leakage. Variable definitions, units and predictor families are summarized in Table 1. The proportion of missing values was small (≤2.8% per variable in the development cohort and ≤7.3% in the external cohort). Baseline characteristics in Table 2 are summarized on observed data before imputation, and the number of missing observations for each variable is reported in a dedicated column; consequently, category counts sum to the cohort total minus the number of missing values rather than to the total itself.

**Table 1:** Definitions, units and predictor families of the 21 source variables (one-hot expanded to 31 model features).

Feature	Type	Unit/levels	Predictor family	Definition
age	Numeric	Years	Demographic	Patient age at enrolment
bmi	Numeric	kg/m^2^	Demographic	Body-mass index
disease_duration_yr	Numeric	Years	Disease history	Time since ulcerative colitis diagnosis
baseline_partial_mayo	Numeric	Score (0–9)	Disease activity	Partial Mayo score at enrolment
fecal_calprotectin	Numeric	μg/g	Biomarker	Faecal calprotectin (mucosal inflammation)
crp_mg_l	Numeric	mg/L	Biomarker	C-reactive protein
esr_mm_h	Numeric	mm/h	Biomarker	Erythrocyte sedimentation rate
hemoglobin_g_dl	Numeric	g/dL	Biomarker	Haemoglobin
albumin_g_l	Numeric	g/L	Biomarker	Serum albumin
platelet_10e9_l	Numeric	10^9^/L	Biomarker	Platelet count
mesalazine_g_d	Numeric	g/day	Medication	Maintenance mesalazine dose
coptis_g	Numeric	g	Herbal dose	Coptidis Rhizoma daily dose
indigo_naturalis_g	Numeric	g	Herbal dose	Indigo naturalis daily dose
sanguisorba_g	Numeric	g	Herbal dose	Sanguisorba daily dose
pulsatilla_g	Numeric	g	Herbal dose	Pulsatilla daily dose
medication_adherence	Numeric	fraction (0–1)	Behavioral	Proportion of prescribed doses taken
distance_to_clinic_km	Numeric	km	Access	Travel distance to treating clinic
sex	Categorical	M/F	Demographic	Biological sex
montreal_extent	Categorical	E1/E2/E3	Disease history	Montreal classification of disease extent
smoking_status	Categorical	Never/Former/Current	Behavioral	Smoking status
tcm_syndrome	Categorical	6 patterns	TCM pattern	Damp-Heat / Spleen-Deficiency / Spleen-Kidney-Yang / Liver-Spleen / Blood-Stasis / Cold-Heat-Complex
relapse_52wk	Binary (target)	0/1	Outcome	Clinical relapse within 52 weeks

**Table 2:** Baseline characteristics of the development cohort and the external Chengdu validation cohort.

Characteristic	Development cohort (*n* = 1,181)	External validation, Chengdu (*n* = 96)	Missing, *n* (Dev / Ext)	*p* value
52-week relapse, *n* (%)	431 (36.5%)	42 (43.8%)	0/0	0.192
Age, years	41.5 ± 13.0	43.5 ± 15.7	26/0	0.113
Female sex, *n* (%)	530 (44.9%)	43 (44.8%)	31/4	0.990
Body-mass index, kg/m^2^	22.5 ± 3.4	22.2 ± 3.5	22/1	0.373
Disease duration, years	3.5 ± 2.3	3.9 ± 2.9	12/5	0.369
Montreal extent E1, *n* (%)	330 (27.9%)	17 (17.7%)	25/3	0.063^a^
Montreal extent E2, *n* (%)	481 (40.7%)	40 (41.7%)	–	–
Montreal extent E3, *n* (%)	345 (29.2%)	36 (37.5%)	–	–
Baseline partial Mayo	2.6 ± 1.6	2.9 ± 1.6	25/3	0.038
Fecal calprotectin, μg/g	235.8 ± 185.7	281.4 ± 271.7	22/1	0.003
C-reactive protein, mg/L	2.5 ± 2.3	2.5 ± 2.1	30/2	0.963
Albumin, g/L	41.1 ± 4.5	40.1 ± 4.9	23/1	0.012
Medication adherence	0.7 ± 0.2	0.7 ± 0.2	28/2	0.003
TCM syndrome: Damp-Heat, *n* (%)	326 (27.6%)	41 (42.7%)	23/2	0.057^b^
TCM syndrome: Spleen-Deficiency, *n* (%)	317 (26.8%)	21 (21.9%)	–	–
TCM syndrome: Spleen-Kidney-Yang, *n* (%)	155 (13.1%)	12 (12.5%)	–	–
TCM syndrome: Liver-Spleen, *n* (%)	190 (16.1%)	10 (10.4%)	–	–
TCM syndrome: Blood-Stasis, *n* (%)	87 (7.4%)	5 (5.2%)	–	–
TCM syndrome: Cold-Heat-Complex, *n* (%)	83 (7.0%)	5 (5.2%)	–	–

Values are presented as mean ± SD for continuous variables and *n* (%) for categorical variables. Observed (pre-imputation) values; percentages over full cohort *n*. Category counts + Missing = cohort total; missing values imputed (median/mode) for modelling only. *p* values by Mann–Whitney U (continuous) or chi-square/Fisher (categorical).

a Montreal-extent.

b TCM-syndrome are overall-distribution tests.

### 3.3 Predictive models and validation

We trained seven supervised classifiers spanning linear, instance-based, kernel, neural and tree-ensemble families: logistic regression, k-nearest neighbours, a support-vector machine with a radial basis function kernel, a multilayer perceptron, random forest, gradient boosting and XGBoost. Each model was wrapped in a standardized preprocessing pipeline so that imputation and scaling were refit inside every fold. Internal performance was estimated by stratified five-fold cross-validation with a fixed seed of 42, preserving the relapse prevalence in each fold. External performance was assessed by refitting each pipeline on the full development cohort and evaluating on the held-out Chengdu cohort. We report AUROC, F1 score, accuracy and the Brier score, and we examine calibration curves and decision-curve net benefit for the selected model (33). AUROC summarizes threshold-independent discrimination, the F1 score and accuracy summarize classification at a chosen operating point, the Brier score jointly captures calibration and refinement, and decision-curve net benefit translates predictions into clinical consequence by weighting false positives and false negatives at a range of threshold probabilities. The selected logistic-regression relapse model, the Brier score and the F1 operating metric are defined in Equations 1–3, respectively. Reporting this full set, rather than AUROC alone, is what allows the predicted probabilities to be used responsibly inside the downstream dosing policy.

$$p=\frac{1}{1+\exp(-(\beta_{0}+\sum_{j=1}^{p}\beta_{j}x_{j}))}$$ (1)

Where p is the predicted 52-week relapse probability, $\beta_{0}$ the intercept, and $\beta_{j}$ the coefficient of standardized feature $x_{j}$ .

$$\mathrm{BS}=\frac{1}{n}\sum_{i=1}^{n}{\left(p_{i}-y_{i}\right)}^{2}$$ (2)

$$\begin{array}{c} F_{1}=\frac{2\cdot \text{Precision}\cdot \text{Recall}}{\text{Precision}+\text{Recall}} \end{array}$$ (3)

Medication adherence was assessed at baseline using the proportion of prescribed medications taken during the preceding 3 months, as documented in outpatient medication records and patient interviews. Adherence was quantified as the medication possession ratio (MPR), calculated as the total number of days covered by prescribed medication divided by the observation period. Values ranged from 0 to 1, with higher values indicating better adherence.

For all machine-learning algorithms, hyperparameters were optimized using a grid-search strategy combined with five-fold cross-validation within the development cohort. Candidate hyperparameter combinations were evaluated according to the mean cross-validated AUROC, and the optimal parameter set was selected using the highest average validation AUROC. For logistic regression, both L1 (LASSO) and L2 (Ridge) regularization penalties were explored, with the inverse regularization strength (C) searched over a logarithmic grid (0.001–100). The final logistic regression model adopted L2 regularization because it achieved the best balance between discrimination and calibration while maintaining model stability across cross-validation folds. To reduce the risk of overfitting, all hyperparameter tuning was performed exclusively within the development cohort. The independent external validation cohort was not used during model development or parameter optimization. Ninety-five percent confidence intervals were computed for all reported performance metrics: for AUROC using the DeLong method, and for the Brier score and the threshold-based metrics (F1, precision, recall, accuracy) using 2,000 stratified bootstrap resamples of the internal out-of-fold and external predictions; DeLong and bootstrap intervals for AUROC were concordant. Calibration was quantified beyond the Brier score by the calibration slope and intercept (from a logistic recalibration of the outcome on the predicted log-odds; ideal values 1 and 0) and the integrated calibration index (ICI); decision-curve net benefit was computed for both the internal and external cohorts against treat-all and treat-none references over a clinically relevant threshold range.

### 3.4 Feature attribution

To understand the drivers of relapse we combined three complementary attribution methods. XGBoost gain quantifies how much each feature improves the splits in which it participates. Permutation importance measures the decrease in AUROC when a feature’s values are randomly shuffled, giving a model-agnostic and validation-based ranking. SHAP (Shapley additive explanations) values decompose individual predictions into additive feature contributions, and their mean absolute value summarizes global importance while preserving the direction of effect (34). Using several methods guards against idiosyncrasies of any single importance measure.

### 3.5 Reinforcement-learning formulation

For the reinforcement-learning framework, herbal treatment intensity was categorized into three predefined herbal intensity tiers (low-, moderate-, and high-intensity) according to the overall prescription strength. The reinforcement-learning algorithm aimed to identify a net-clinical-benefit policy, defined as the treatment policy that maximizes the expected long-term clinical reward while balancing relapse prevention against treatment burden. We recast remission maintenance as a sequential decision problem over scheduled follow-up visits, yielding 4,141 state-action-reward transitions. The state at each visit was the patient’s current inflammatory and nutritional profile (fecal calprotectin, partial Mayo score, C-reactive protein, albumin, haemoglobin and medication adherence). The action was the herbal-intensity tier administered until the next visit, discretized into three levels: Reduced, Standard and Intensified. The reward encoded the gain in remission status at the next visit. Because randomized exploration is not feasible in routine herbal maintenance, we used model-based off-policy evaluation: a gradient-boosted reward (Q) model was fit to predict the expected reward for each state-action pair, enabling estimation of the value of candidate policies that were never actually deployed. The discounted return that a policy seeks to maximize and the model-based estimate of a policy’s value are defined in Equations 4, 5.

$$\begin{array}{c} G_{t}=\sum_{k=0}^{T}\gamma^{k}r_{t+k} \end{array}$$ (4)

$$\begin{array}{c} V(\pi )=E[Q(s,\pi (s))] \end{array}$$ (5)

The optimized policy maximizes a net-clinical-benefit objective rather than expected reward alone. Specifically, the objective is the expected reward minus a treatment-burden term that is scaled by disease quiescence, so that intensifying a patient who is already quiescent is penalized; this net-clinical-benefit objective is defined in Equation 6. This design directly encodes the clinical reality that over-treatment carries costs: polypharmacy, expense and the safety-monitoring burden associated with agents such as Indigo naturalis. We compared the learned net-benefit policy against the observed clinician (behavior) policy, a random policy, and three fixed policies (Always Reduced, Always Standard, Always Intensified), and we examined how the recommended action distribution varied with baseline calprotectin and whether concordance with the policy was associated with next-visit remission.

$$\begin{array}{c} \mathrm{NB}(s,a)=Q(s,a)-\kappa \cdot b(a)\cdot g(s) \end{array}$$ (6)

Where Q(s,a) is the expected reward of action a in state s , b(a) the treatment-burden weight, g(s) a disease-quiescence term, and κ a tunable penalty coefficient.

## 4 Results

### 4.1 Cohort composition and feature structure

Table 2 summarizes the two cohorts. The development cohort comprised 1,181 analysable patients with a 52-week relapse rate of 36.5%, while the external Chengdu cohort comprised 96 patients with a higher relapse rate of 43.8%. The cohorts were broadly comparable in age (41.5 +/− 13.0 versus 43.5 +/− 15.7 years), sex distribution (44.9% versus 44.8% female), body-mass index and disease duration, supporting their use as a development-validation pair. Two differences are clinically relevant for transportability: the external cohort had a larger share of extensive disease (Montreal E3 37.5% versus 29.2%) and higher mean fecal calprotectin (281.4 versus 235.8 ug/g), consistent with its higher observed relapse rate. The distribution of TCM syndromes also differed, with Damp-Heat more prevalent at the external site (42.7% versus 27.6%), reflecting genuine case-mix shift between centres. Formal between-cohort comparisons (Table 2, *p*-value column) confirmed that the cohorts were statistically similar in age (*p* = 0.11), sex (*p* = 0.99), body-mass index (*p* = 0.37), disease duration (p = 0.37), C-reactive protein (*p* = 0.96) and the 52-week relapse rate (*p* = 0.19). The external Chengdu cohort nevertheless differed on markers of disease burden, with significantly higher fecal calprotectin (*p* = 0.003) and baseline partial Mayo score (*p* = 0.038), lower serum albumin (*p* = 0.012) and lower medication adherence (p = 0.003), together with borderline differences in Montreal disease extent (*p* = 0.063) and TCM-syndrome distribution (*p* = 0.057). This pattern is consistent with a modestly higher-risk external population and confirms a genuine case-mix shift, strengthening the external cohort as a test of transportability.

Table 1 defines all 21 source variables, their units and the predictor family to which they belong (demographic, disease history, biomarker, herbal dose, behavioral, access, TCM pattern and outcome). After one-hot encoding of the four categorical variables, the model operated on 31 features. Grouping variables into families clarifies the later attribution results, in which behavioral (adherence), biomarker (calprotectin, CRP, albumin) and herbal-dose (Coptidis Rhizoma) families dominate.

**Figure 1:**
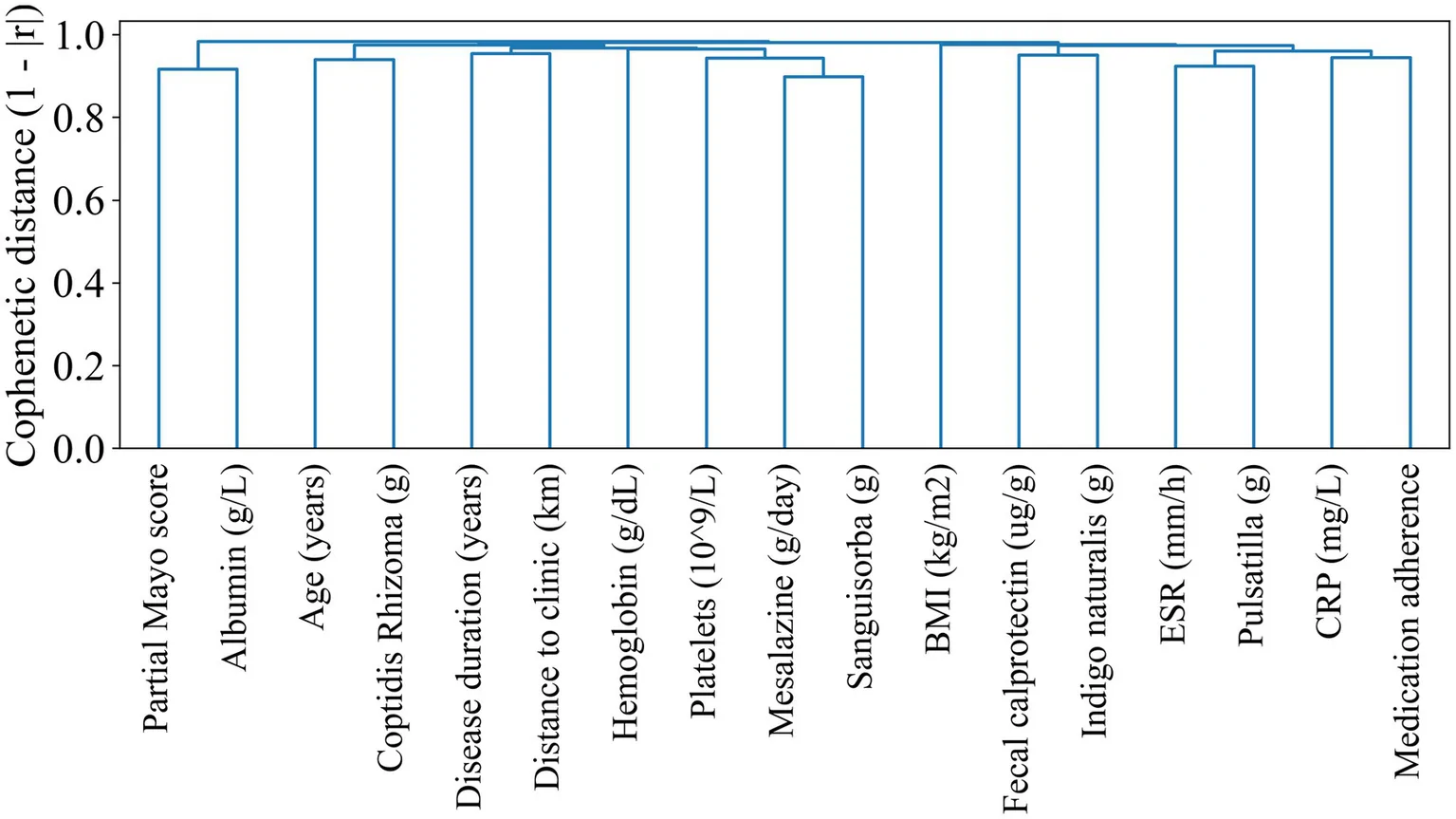
Hierarchical-clustering dendrogram of clinical and herbal features using a Spearman-correlation distance, showing coherent biomarker, herbal-dose and demographic feature blocks.

Figure 1 shows a hierarchical-clustering dendrogram of the clinical and herbal features computed with a Spearman-correlation distance. The dendrogram reveals coherent blocks rather than a single undifferentiated mass: inflammatory biomarkers cluster together, the herbal-dose variables form an adjacent block, and demographic and access variables separate on their own branches. The absence of near-zero-distance merges indicates that the predictors carry largely non-redundant information, which justifies retaining the full feature set rather than aggressively pruning correlated variables before modelling.

### 4.2 Outcome distribution and clinical correlates

**Figure 2:**
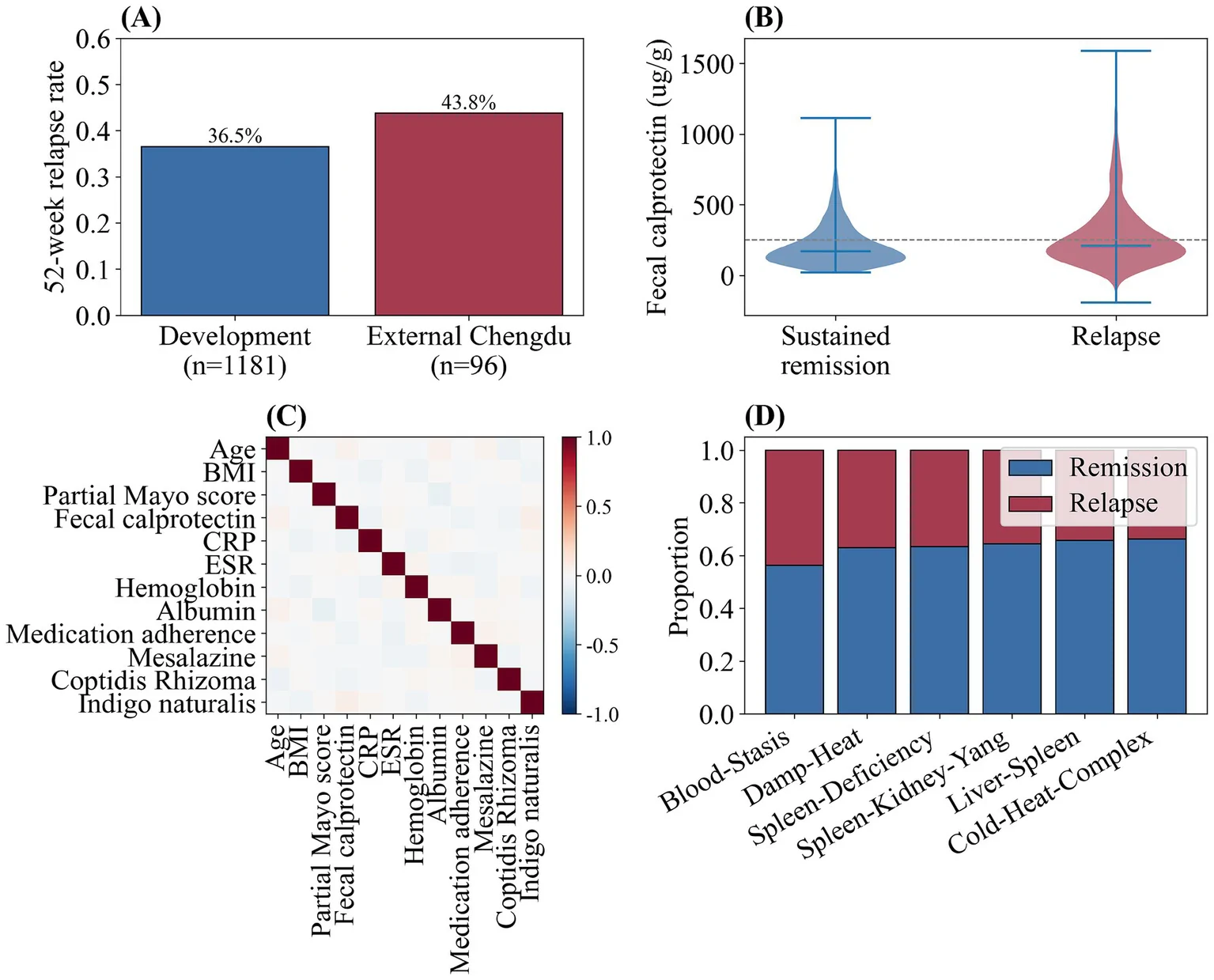
**(A)** Relapse prevalence by cohort; **(B)** Fecal calprotectin by outcome with a 250 ug/g reference line; **(C)** Feature correlation heatmap; **(D)** Relapse rate by TCM syndrome.

Figure 2 characterizes the relapse outcome and its clinical correlates. Figure 2A compares outcome prevalence across cohorts, confirming the 36.5% relapse rate in the development cohort and the 43.8% rate in the external Chengdu cohort; the moderate class imbalance (relapse being the minority class) motivates our emphasis on F1 score and threshold tuning in addition to AUROC. Figure 2B shows the distribution of fecal calprotectin by outcome as violin plots, with a dashed reference line at 250 ug/g; patients who relapsed had visibly higher calprotectin, and a substantial share exceeded the 250 ug/g threshold that is widely used as a signal of subclinical mucosal inflammation (35, 36), foreshadowing the central role calprotectin plays in both prediction and the dosing policy. Figure 2C presents the feature correlation heatmap, which mirrors the dendrogram: inflammatory biomarkers correlate positively with one another and inversely with albumin, while herbal-dose variables form a weakly correlated block, again indicating limited redundancy. Figure 2D displays relapse rates by TCM syndrome as stacked bars; relapse fractions ranged from roughly 0.34 to 0.44 across the six syndromes, with Blood-Stasis showing the highest relapse share (about 0.44) and Cold-Heat-Complex the lowest (about 0.34). The modest spread suggests that syndrome label alone is a weak univariate predictor, consistent with its low downstream attribution and supporting the need for multivariable, biomarker-driven modelling.

### 4.3 Unsupervised structure

**Figure 3:**
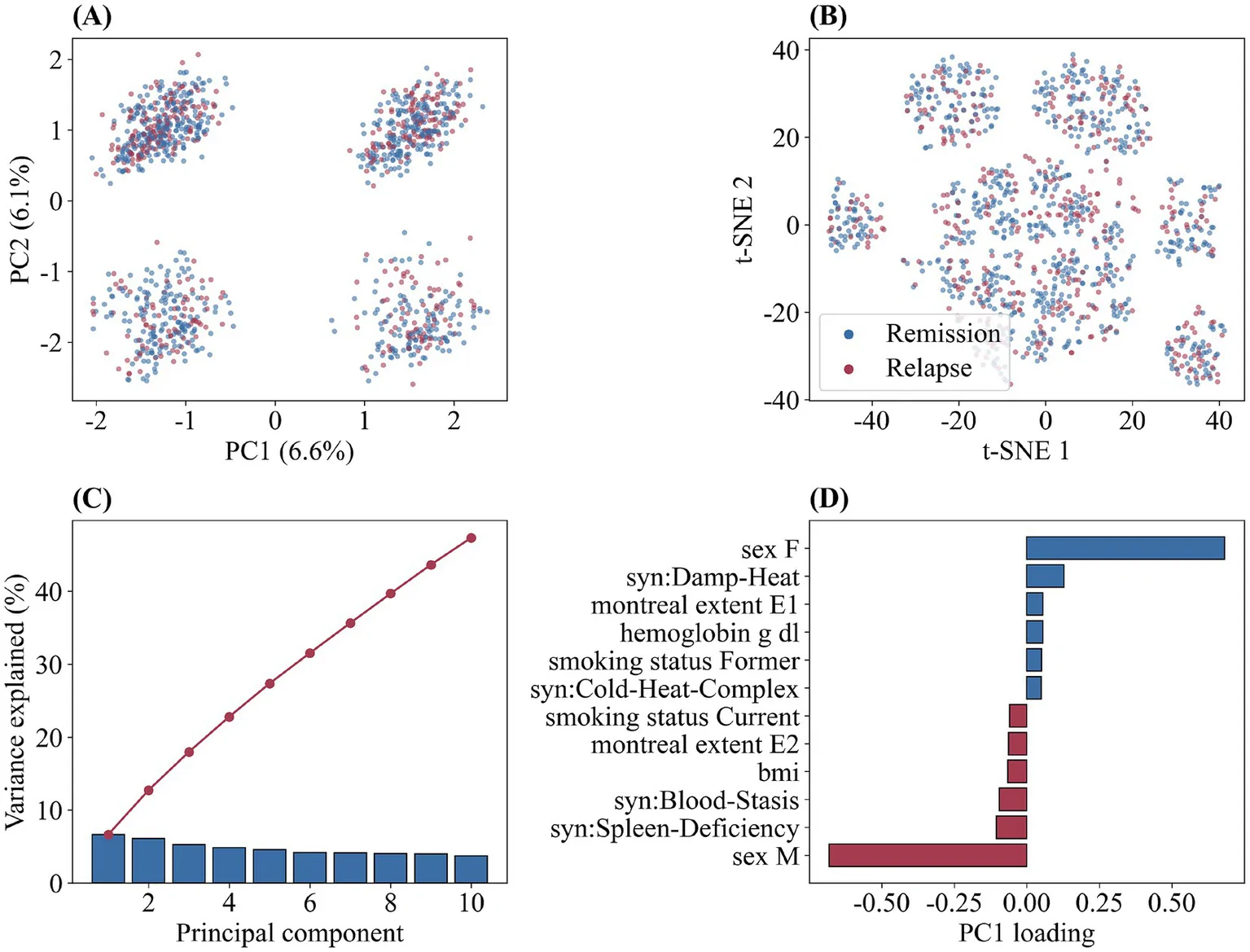
**(A)** PCA projection by outcome; **(B)** t-SNE embedding by outcome; **(C)** scree plot with cumulative explained variance; **(D)** leading PC1 feature loadings.

Before supervised modelling we examined the unsupervised structure of the feature space (Figure 3). Figure 3A projects patients onto the first two principal components coloured by outcome, and Figure 3B shows the corresponding t-SNE embedding. In both projections, patients who relapsed and those who remained in remission overlap heavily without forming separated clusters. This is an informative negative result: it indicates that relapse risk is not encoded along a single dominant axis of variation and is instead a subtle, multivariable signal, which is exactly the regime in which supervised models add value over simple stratification. Figure 3C, the scree plot with cumulative variance, reinforces this interpretation: the leading principal component explains only about 6.6% of variance and the first ten components together explain under 50%, so no small set of latent factors captures the data. Figure 3D shows the leading PC1 loadings, which are spread across inflammatory biomarkers and herbal-dose variables rather than being dominated by a single feature, consistent with the diffuse variance structure. Together these panels argue that dimensionality reduction is poorly suited to this problem and that a flexible but regularized supervised model is the appropriate tool.

### 4.4 Model benchmarking and external validation

**Figure 4:**
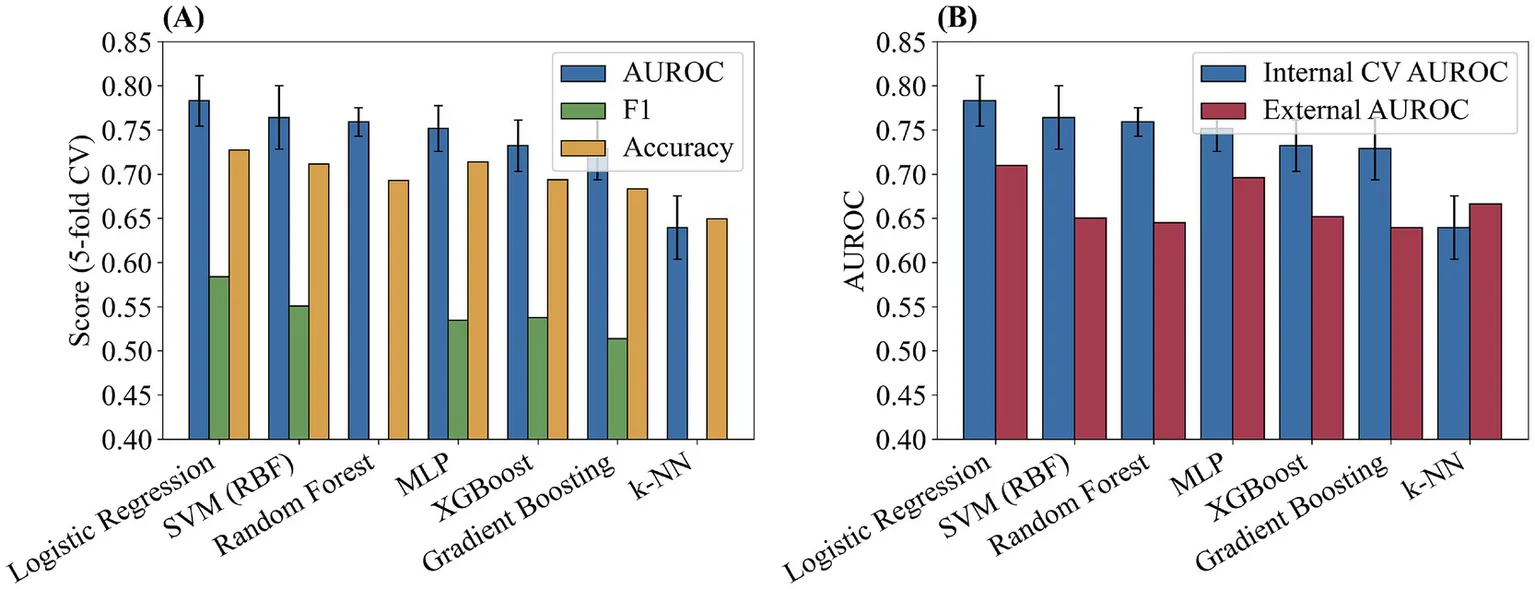
**(A)** Internal five-fold cross-validation AUROC, F1 and accuracy across seven models; **(B)** Internal versus external AUROC per model.

Figure 4 and Table 3 compare the seven candidate models. Figure 4A presents internal five-fold cross-validation AUROC, F1 and accuracy as grouped bars across the models. Logistic regression achieved the highest cross-validation AUROC (0.783 ± 0.029; 95% CI 0.755–0.809), followed closely by the RBF support-vector machine (0.764) and random forest (0.759), with k-NN trailing (0.639). The tree ensembles did not outperform the linear model, which is typical when the sample size is moderate and the signal is approximately additive. Figure 4B plots internal versus external AUROC for each model and is the more decision-relevant panel: it exposes how much performance is lost under distribution shift. Logistic regression retained the best external AUROC among the strong internal models (0.710; 95% CI 0.600–0.820), whereas the SVM and random forest dropped more sharply (to 0.650 and 0.645, respectively). Some models that were mediocre internally, such as the MLP (external 0.696) and k-NN (external 0.666), held up relatively well externally, but neither matched the combination of internal and external performance achieved by logistic regression. On the basis of this internal-plus-external profile, together with its favourable Brier score (0.180, 95% CI 0.169–0.191, internal; 0.215, 95% CI 0.170–0.263, external) and transparency, we selected logistic regression as the primary model for all subsequent analyses.

**Table 3:** Internal five-fold cross-validation and external-validation performance of the seven candidate models, ordered by cross-validation AUROC.

Model	CV AUROC (mean ± SD)	CV F1	CV accuracy	CV Brier	External AUROC	External F1	External accuracy	External Brier
Logistic Regression	0.783 ± 0.029	0.584	0.727	0.18	0.71	0.615	0.688	0.215
SVM (RBF)	0.764 ± 0.036	0.551	0.711	0.189	0.65	0.608	0.677	0.232
Random Forest	0.759 ± 0.016	0.378	0.693	0.198	0.645	0.459	0.656	0.228
MLP	0.752 ± 0.026	0.534	0.714	0.193	0.696	0.522	0.656	0.216
XGBoost	0.732 ± 0.029	0.537	0.693	0.206	0.652	0.532	0.615	0.248
Gradient Boosting	0.729 ± 0.035	0.513	0.683	0.204	0.639	0.593	0.656	0.247
k-NN	0.639 ± 0.036	0.255	0.649	0.221	0.666	0.407	0.635	0.231

### 4.5 Learning behavior and hyperparameter sensitivity

**Figure 5:**
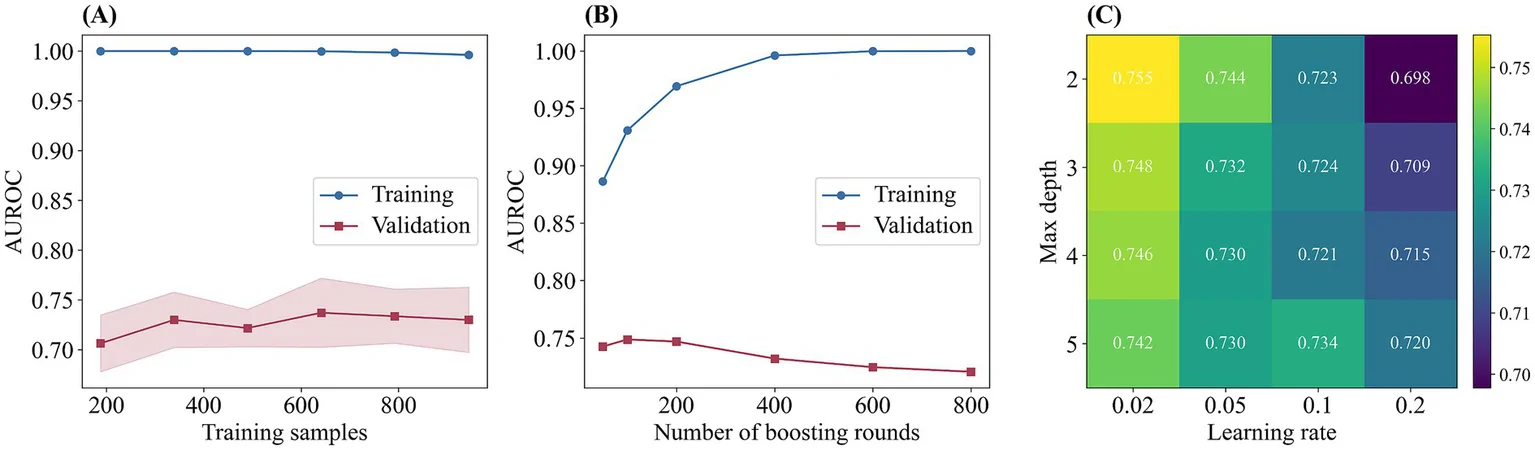
**(A)** Learning curve of AUROC versus training-set size; **(B)** validation curve over boosting rounds; **(C)** hyperparameter sensitivity heatmap (maximum depth versus learning rate, cross-validation AUROC).

Figure 5 probes whether the models were data-limited or mis-tuned. Figure 5A is a learning curve of AUROC against training-set size; the validation AUROC rises steeply at small sample sizes and then plateaus, with the training and validation curves converging. The plateau indicates that the model is in a low-variance regime where adding more patients yields diminishing returns, so the moderate performance reflects intrinsic signal difficulty rather than insufficient data. Figure 5B is a validation curve over boosting rounds for the gradient-boosted model; validation AUROC peaks at a modest number of rounds and then declines as additional rounds overfit, confirming the value of early stopping and regularization. Figure 5C is a hyperparameter-sensitivity heatmap of cross-validation AUROC over a grid of maximum tree depth and learning rate; performance is highest in a broad region of shallow trees with moderate learning rates and degrades for deep trees, showing that the tuned configuration sits on a stable plateau rather than a fragile peak. Collectively, Figure 5 indicates that the benchmark in Figure 4 is a fair reflection of each model’s capacity on this task.

### 4.6 Discrimination and classification of the selected model

**Figure 6:**
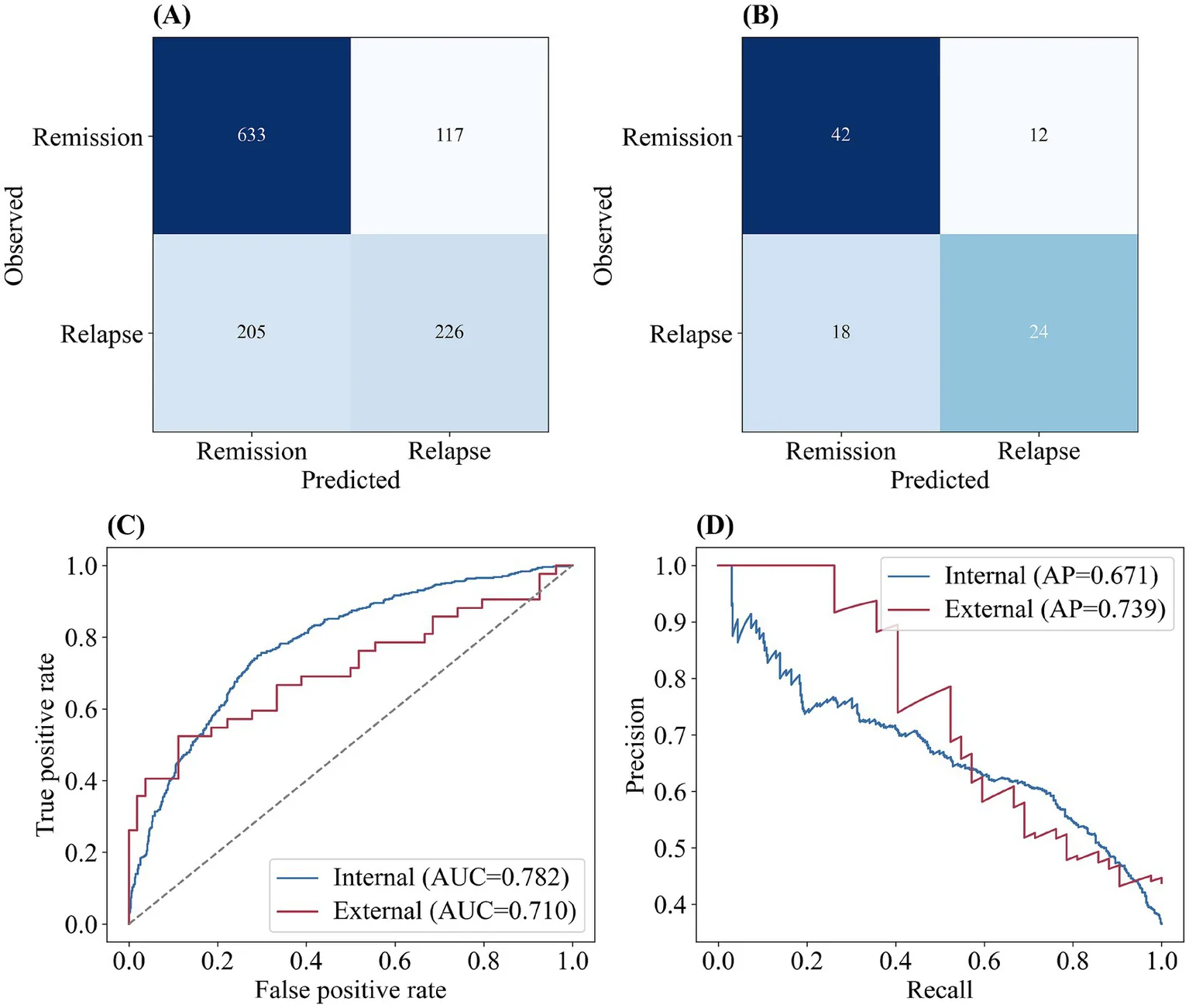
**(A)** Internal out-of-fold confusion matrix; **(B)** External confusion matrix; **(C)** Internal versus external ROC curves; **(D)** Internal versus external precision-recall curves.

Figure 6 and Table 4 detail the performance of the selected logistic-regression model. Figure 6A is the internal confusion matrix computed from out-of-fold predictions, and Figure 6B is the external confusion matrix on the Chengdu cohort; both are dominated by correct classifications but show the expected trade-off in which a minority of relapsers are missed and a minority of non-relapsers are flagged, appropriate for a screening-style risk tool. Figure 6C overlays the internal and external ROC curves, visually confirming the AUROC values of 0.783 (95% CI 0.755–0.809) internally and 0.710 (95% CI 0.600–0.820) externally; the external curve lies below the internal curve but well above the diagonal, demonstrating preserved discrimination under distribution shift. Figure 6D shows the internal and external precision-recall curves, which are more informative under class imbalance; the model maintains useful precision across a wide recall range, supporting its use for prioritizing higher-risk patients. Table 4 consolidates these operating characteristics, including the threshold-0.32 operating point discussed below: at this threshold the model attains an F1 of 0.663 with precision 0.592 and recall 0.754, favouring sensitivity so that fewer impending relapses are missed.

**Table 4:** Operating characteristics of the selected logistic-regression model, internal versus external.

Metric	Internal (5-fold CV/OOF)	External (Chengdu)
AUROC	0.783 (95% CI 0.755–0.809)	0.710 (95% CI 0.600–0.820)
F1 (0.50 cut-off)	0.584 (0.544–0.625)	0.615 (0.480–0.734)
Accuracy (0.50 cut-off)	0.727 (0.704–0.750)	0.688 (0.594–0.771)
Brier score	0.180 (0.169–0.191)	0.215 (0.170–0.263)
Precision @ 0.32	0.592 (0.564–0.622)	—
Recall @ 0.32	0.754 (0.712–0.796)	—
F1 @ 0.32	0.663 (0.634–0.692)	—

### 4.7 Drivers of relapse

**Figure 7:**
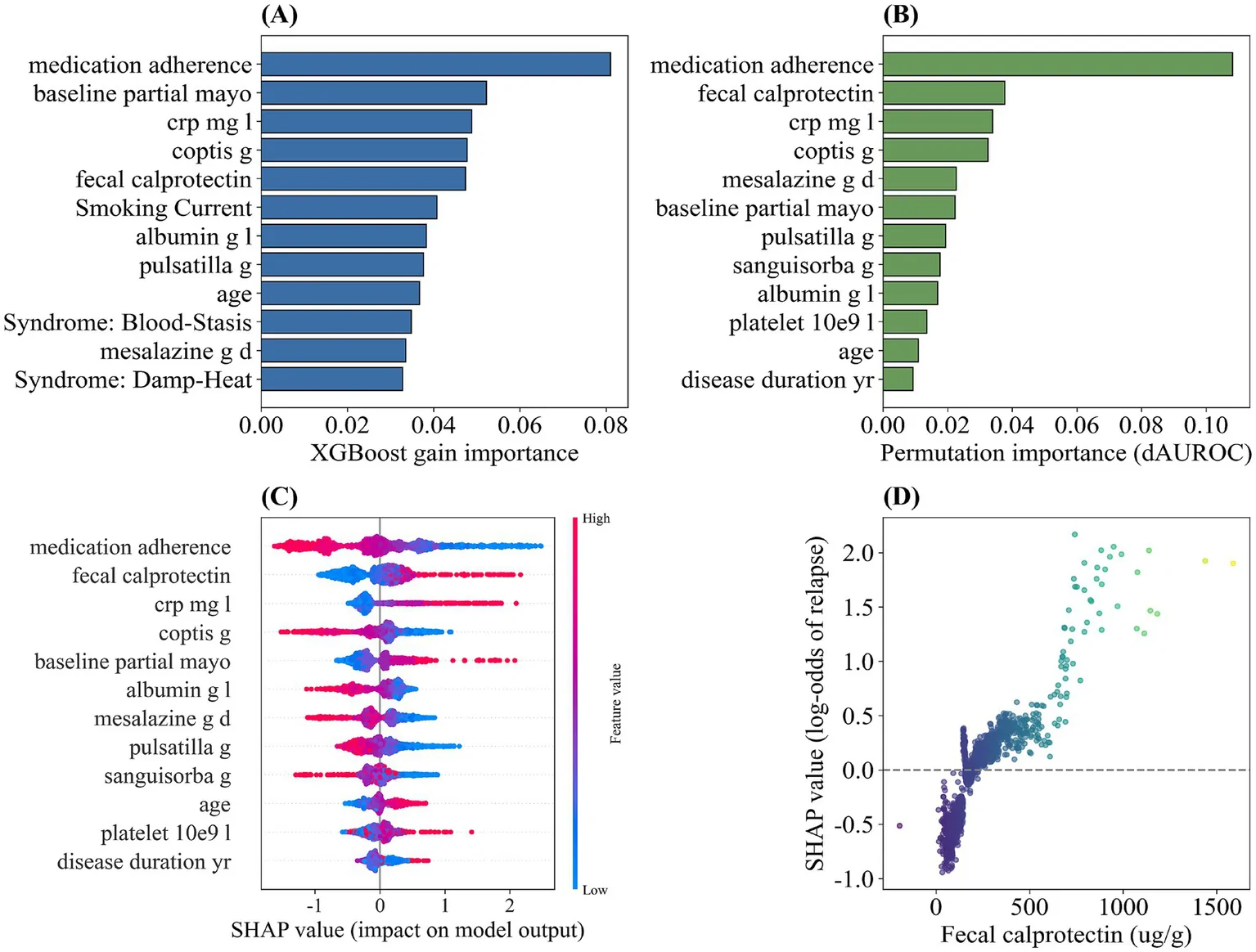
**(A)** XGBoost gain importance; **(B)** Permutation importance (delta-AUROC); **(C)** SHAP beeswarm summary; **(D)** SHAP dependence for fecal calprotectin, showing a monotonic increase in relapse log-odds.

Figure 7 and Table 5 summarize the principal drivers of relapse using three complementary feature-attribution approaches. XGBoost gain importance (Figure 7A) quantifies the contribution of each variable to tree splitting during model training, permutation importance (Figure 7B) evaluates the reduction in AUROC after randomly shuffling individual variables, and the SHAP beeswarm plot (Figure 7C) illustrates the magnitude and direction of each feature’s contribution to the predicted relapse risk. Overall, the three approaches identified highly consistent patterns, supporting the robustness of the identified predictors. Medication adherence was consistently the most influential predictor, exhibiting the highest mean absolute SHA*p* value (0.699), the greatest XGBoost gain (0.081), and the largest permutation importance (∆AUROC = 0.108), indicating that treatment adherence contributed substantially more to predictive performance than any other variable. Fecal calprotectin (mean |SHAP| = 0.358), C-reactive protein (0.347), and Coptidis Rhizoma dose (0.341) formed the second tier of influential predictors across all attribution methods. Baseline partial Mayo score (0.311), albumin (0.284), mesalazine dose (0.282), Pulsatilla dose (0.252), Sanguisorba dose (0.182), age (0.173), platelet count (0.154), and disease duration (0.132) also contributed to model prediction, although their permutation importance was comparatively smaller (Figure 7B and Table 5). Notably, the inclusion of multiple herbal-dose variables among the top-ranked predictors suggests that prescription composition itself carries important prognostic information and provides a biological rationale for the subsequent reinforcement-learning-based dose optimization. The SHAP dependence plot for fecal calprotectin (Figure 7D) further demonstrated a clear positive relationship between fecal calprotectin concentration and the predicted log-odds of relapse. Increasing fecal calprotectin values were associated with progressively higher SHA*p* values, indicating a monotonic increase in predicted relapse risk and providing an interpretable biological explanation for one of the model’s strongest predictors. Table 5 presents the top 12 predictors together with their corresponding mean absolute SHAP values, XGBoost gain importance, and permutation ∆AUROC values.

**Table 5:** Top-12 predictors ranked by mean absolute SHAP value, with XGBoost gain and permutation delta-AUROC for cross-reference.

Rank	Feature	Mean |SHAP|	XGBoost gain	Permutation ΔAUROC
1	medication_adherence	0.699	0.081	0.108
2	fecal_calprotectin	0.358	0.047	0.038
3	crp_mg_l	0.347	0.049	0.034
4	coptis_g	0.341	0.048	0.032
5	baseline_partial_mayo	0.311	0.052	0.022
6	albumin_g_l	0.284	0.038	0.017
7	mesalazine_g_d	0.282	0.033	0.023
8	pulsatilla_g	0.252	0.038	0.019
9	sanguisorba_g	0.182	0.031	0.018
10	age	0.173	0.037	0.011
11	platelet_10e9_l	0.154	0.027	0.014
12	disease_duration_yr	0.132	0.027	0.009

Among all predictors, medication adherence emerged as the strongest determinant of relapse risk across all feature-attribution methods. Importantly, unlike demographic characteristics or disease duration, medication adherence represents a readily modifiable risk factor in routine clinical practice. Poor adherence has consistently been associated with increased relapse rates in patients with ulcerative colitis and may be improved through patient education, digital adherence monitoring, medication reminders, pharmacist-led interventions, and regular follow-up. Therefore, identifying patients with poor adherence provides clinicians with an immediate opportunity for intervention before escalation to more intensive therapies. Similarly, fecal calprotectin and C-reactive protein are objective inflammatory biomarkers that are routinely monitored during remission maintenance. Their strong predictive importance supports the biological plausibility of the model and suggests that serial measurement of these biomarkers may facilitate earlier identification of patients at increased risk of relapse. Consequently, the leading predictors identified by the model are not only statistically important but also clinically actionable, supporting their integration into personalized decision-support systems.

### 4.8 Calibration, threshold tuning and clinical utility

**Figure 8:**
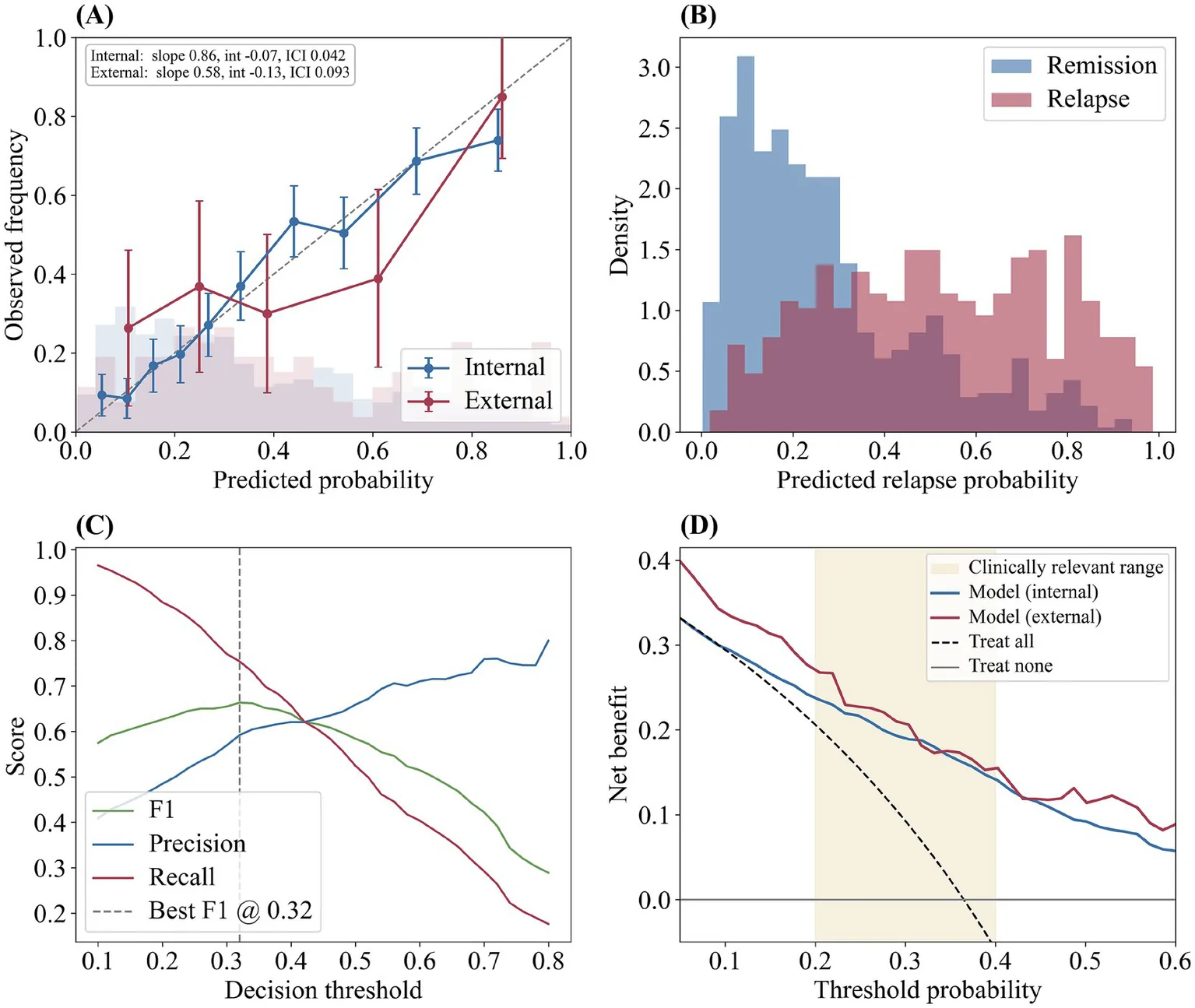
**(A)** Calibration is shown as binned observed-versus-predicted relapse probability with 95% confidence intervals and an overlaid histogram of predicted risk; calibration slope, intercept, and the integrated calibration index (*ICI*) are annotated for both cohorts; **(B)** Risk-score separation histogram; **(C)** Threshold tuning of *F1*, precision, and recall (best *F1* at 0.32); **(D)** Decision-curve net benefit is plotted for the internal and external cohorts against treat-all and treat-none references, with the clinically relevant threshold range (0.20–0.40) shaded.

Figure 8 evaluates whether the model’s probabilities are trustworthy and clinically useful. Figure 8A shows calibration curves for the internal and external cohorts against the diagonal of perfect calibration; both curves track the diagonal closely, and the low Brier scores (0.180 internal, 0.215 external) confirm that the predicted probabilities are well calibrated rather than merely rank-discriminative, which is essential when probabilities feed a decision policy. Figure 8B is a histogram of the risk score stratified by true outcome, showing meaningful separation between relapsers and non-relapsers despite the overlap expected from a moderate-AUROC task. Figure 8C plots F1, precision and recall against the decision threshold; F1 is maximized at a threshold of 0.32 (F1 0.663, precision 0.592, recall 0.754), well below the default 0.50, reflecting the minority status of relapse and the clinical preference for sensitivity in a maintenance-monitoring setting. Figure 8D is a decision-curve analysis showing net benefit across a range of threshold probabilities; the model provides positive net benefit over a clinically relevant range and exceeds both the treat-all and treat-none strategies, the metric most directly tied to clinical decision-making and the one we later embed in the reinforcement-learning objective. The predicted probabilities were well calibrated: the internal calibration slope was 0.86 with an intercept of −0.07 and an ICI of 0.042, and the mean predicted risk (0.365) matched the observed relapse rate (0.365). Externally, calibration-in-the-large remained good (mean predicted 0.445 vs. observed 0.438), although the calibration slope of 0.58 (intercept −0.13, ICI 0.093) indicates that predictions were modestly too extreme under distribution shift, a limitation we now display explicitly with binned observed-versus-predicted points and 95% confidence intervals in Figure 8A. The decision-curve analysis (Figure 8D), now shown for both cohorts with the clinically relevant threshold range (0.20–0.40) shaded, confirms positive net benefit exceeding both the treat-all and treat-none strategies across that range.

### 4.9 Reinforcement-learning policy for herbal-intensity titration

**Figure 9:**
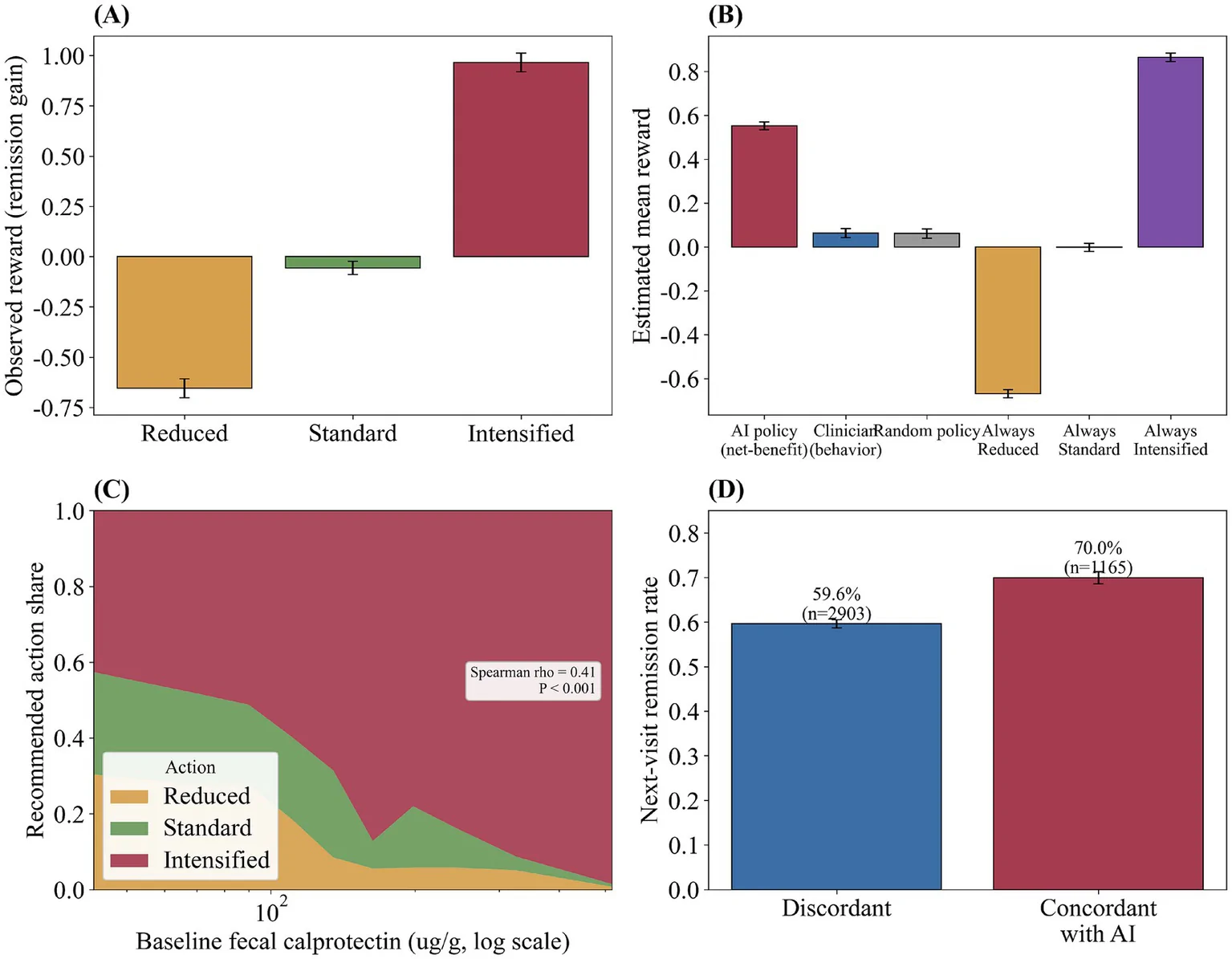
**(A)** Observed reward by administered action; **(B)** Off-policy value estimates of candidate policies; **(C)** Recommended herbal-intensity action share (Reduced/Standard/Intensified) as a continuous stacked-area distribution across baseline fecal calprotectin (deciles), illustrating the monotonic escalation of recommended intensity with rising calprotectin (Spearman *ρ* = 0.41); **(D)** Next-visit remission rate by concordance with the AI policy.

Figure 9 and Table 6 present the reinforcement-learning analysis built on 4,141 scheduled-visit transitions. Figure 9A shows the observed reward by administered action (Reduced, Standard, Intensified), establishing the behavior-policy baseline; on average, more intensive herbal dosing was associated with greater observed remission gain, but this raw association is confounded by indication because sicker patients are intensified more often. Figure 9B reports model-based off-policy value estimates for six policies. The naive Always Intensified policy attains the highest raw estimated reward (0.864 +/− 0.019), but this ignores treatment burden and would expose quiescent patients to unnecessary polypharmacy. The net-clinical-benefit AI policy achieves a high yet burden-aware reward (0.552 +/− 0.018), far exceeding the observed clinician behavior policy (0.063 +/− 0.021) and a random policy (0.061 +/− 0.022), while Always Reduced is harmful (−0.669 +/− 0.018) and Always Standard is near zero (−0.002 +/− 0.019). The gap between the AI policy and clinician behavior quantifies the headroom for improvement from systematic, state-dependent dosing. Figure 9C shows how the personalized policy adapts to disease activity by reporting the recommended action share within baseline calprotectin tertiles. In the low-calprotectin tertile the policy recommends a more balanced mix (Reduced 28.4%, Standard 24.2%, Intensified 47.4%), preserving the option to de-escalate quiescent patients. As calprotectin rises, the policy shifts decisively toward intensification: 76.0% Intensified in the mid tertile and 89.4% in the high tertile, with the Reduced share falling to 3.9%. This monotonic gradient is exactly the behavior the net-benefit objective was designed to produce - sparing quiescent patients while escalating those with active subclinical inflammation - and it mirrors the SHAP dependence of calprotectin in Figure 7D. Figure 9D links the policy to outcomes by comparing next-visit remission rates according to whether actual care was concordant with the AI recommendation: concordant visits (n = 1,165) had a remission rate of 70.0%, versus 59.6% for discordant visits (n = 2,903), a 10.4-percentage-point absolute difference. Although observational, this association indicates that the policy captures actionable information rather than merely fitting noise. Beyond the tertile summary, the recommended-action share varied smoothly and monotonically across baseline fecal calprotectin (Figure 9C, now shown as a continuous stacked-area distribution over calprotectin deciles): the Intensified share rose from 42.7% in the lowest decile (median calprotectin 43 μg/g) to 98.6% in the highest (median 516 μg/g), while the Reduced share fell from 30.4 to 0.7%. The recommended herbal-intensity tier correlated positively with baseline calprotectin (Spearman *ρ* = 0.41, *p* < 0.001), quantifying the calprotectin-graded escalation that the net-benefit objective was designed to produce. Table 6 consolidates the off-policy value estimates and the concordance results.

**Table 6:** Off-policy value estimates of candidate policies (estimated mean reward +/− SEM) and next-visit remission rate by concordance with the AI policy.

Policy/comparison	Estimated mean reward (± SEM)	Next-visit remission rate	*n*
AI policy (net-benefit)	0.552 ± 0.018	–	–
Clinician (behavior)	0.063 ± 0.021	–	–
Random policy	0.061 ± 0.022	–	–
Always reduced	−0.669 ± 0.018	–	–
Always standard	−0.002 ± 0.019	–	–
Always intensified	0.864 ± 0.019	–	–
Concordant with AI policy	–	70.0% ± 1.3%	1,165
Discordant with AI policy	–	59.6% ± 0.9%	2,903

## 5 Discussion

This study shows that interpretable prediction and reinforcement learning can be combined to personalize herbal-intensity decisions in ulcerative colitis remission maintenance. Three findings stand out. First, a calibrated logistic-regression model predicts 52-week relapse with good discrimination internally (AUROC 0.783, 95% CI 0.755–0.809) and retains transportable performance on an independent external cohort (AUROC 0.710, 95% CI 0.600–0.820) (25). Second, the drivers of relapse are dominated by medication adherence, followed by objective inflammatory markers (fecal calprotectin, C-reactive protein) and, importantly, a herbal-dose variable (Coptidis Rhizoma), linking the prescription itself to outcome. Third, a net-clinical-benefit reinforcement-learning policy escalates herbal intensity in proportion to disease activity and is associated with a higher next-visit remission rate when actual care agrees with it.

The clinical reading of these results is coherent. Adherence emerging as the single strongest predictor (30, 31) reframes part of the relapse problem as an adherence problem and suggests that adherence support may be as valuable as pharmacological intensification for some patients. The monotonic SHAP dependence of calprotectin (Figure 7D) and the calprotectin-graded action shares of the policy (Figure 9C) tell a single consistent story: subclinical mucosal inflammation, captured by calprotectin, should drive both risk assessment and dosing. The policy operationalizes this by recommending de-escalation or maintenance for quiescent patients and decisive intensification for those with rising calprotectin, in contrast to fixed regimens that hold intensity constant regardless of inflammatory trajectory.

Our framing also clarifies why the burden-aware objective matters. A naive policy that always intensifies attains the highest raw estimated reward (Figure 9B), but it would impose polypharmacy, cost and safety-monitoring burden–particularly relevant for Indigo naturalis–on patients who do not need it (24). By subtracting a treatment-burden term scaled by disease quiescence, the net-benefit policy retains most of the achievable benefit while avoiding indiscriminate escalation, which is the clinically deployable behavior. The association between policy concordance and higher remission (70.0% versus 59.6%) provides a tangible, interpretable signal of potential value that is easier for clinicians to act on than an abstract reward estimate.

Compared with prior decision-support work for IBD, which has emphasized one-shot relapse prediction (9, 37), our contribution is to treat maintenance as the sequential problem it is and to evaluate the resulting policy with model-based off-policy estimation, calibration and decision-curve net benefit rather than discrimination alone (16, 33). The choice of a transparent linear model as the deployed predictor, with nonlinear models reserved for attribution, balances performance against the interpretability that clinical adoption requires.

The clinical implications are concrete. A fixed herbal maintenance regimen, by construction, applies the same intensity to a patient whose calprotectin is quietly climbing and to a patient who is durably quiescent; our results suggest that both groups are mismanaged by such a policy–the former under-treated and at elevated relapse risk, the latter over-treated and exposed to avoidable burden. A practical deployment would surface, at each visit, the model’s calibrated relapse probability together with the policy’s recommended intensity tier, allowing the clinician to weigh escalation against burden in the context of the patient’s adherence and biomarker trajectory, consistent with treat-to-target maintenance goals that incorporate normalization of fecal calprotectin (38). Because the deployed predictor is a linear model with explicit coefficients and the policy’s action shares vary smoothly with calprotectin, the recommendation can be explained to the patient in terms they can act on, which is itself a determinant of adherence–the very factor that the attribution analysis identified as the strongest driver of relapse. The 10.4-percentage-point remission difference associated with policy concordance, while observational, is of a magnitude that would be clinically meaningful if even partially causal, and it sets a clear effect size for a future prospective trial to target.

An important observation from the external validation was the presence of a moderate case-mix shift between the development and Chengdu cohorts. Compared with the development cohort, patients in the external cohort exhibited a higher 52-week relapse rate, a greater proportion of extensive disease (Montreal E3), higher baseline fecal calprotectin concentrations, and a different distribution of traditional Chinese medicine (TCM) syndrome patterns, particularly a higher prevalence of Damp-Heat syndrome. These differences likely reflect variations in referral patterns, regional disease characteristics, and clinical practice across institutions. Importantly, despite these baseline differences, the prediction model maintained good discrimination and calibration in the external cohort, suggesting that it captured prognostic relationships that were not restricted to the development population. Nevertheless, the observed case-mix shift indicates that model performance may vary across populations with different clinical characteristics or TCM syndrome distributions. Therefore, further validation in larger multicentre cohorts representing broader geographic regions and more diverse patient populations will be essential to confirm the model’s generalizability and to determine whether regional recalibration or model updating is required before widespread clinical implementation.

Several limitations of the reinforcement-learning framework should be acknowledged. First, off-policy evaluation relied on a model-based reward estimator rather than prospective evaluation under the learned treatment policy. Consequently, the estimated policy value depends on the accuracy of the gradient-boosted reward model and may be affected by model misspecification or residual confounding. Second, because reinforcement learning was trained using retrospective observational data, the estimated rewards for treatment strategies that were rarely or never observed rely on counterfactual prediction rather than direct clinical evidence. To minimize this risk, the recommended treatment actions were restricted to clinically observed herbal intensity tiers, thereby reducing extrapolation beyond the empirical data distribution. Nevertheless, some degree of distributional shift between historical treatment patterns and the learned policy cannot be completely excluded. Therefore, the reinforcement-learning policy should be regarded as hypothesis-generating and requires prospective validation in randomized or pragmatic clinical studies before routine clinical implementation.

Several limitations temper these conclusions. The analysis is observational, so the off-policy value estimates and the concordance-remission association are vulnerable to confounding by indication and to unmeasured factors; concordant care may be a marker of otherwise better-managed patients rather than a cause of remission. Model-based off-policy evaluation inherits the assumptions of the gradient-boosted reward model and assumes adequate overlap between the behavior and evaluated policies, which can be violated in regions the clinicians rarely explored (20, 21). External validation, although a genuine strength, rests on a single site (Chengdu) of modest size (*n* = 96), and the higher relapse rate and case-mix shift there caution against assuming wider generalizability. The herbal-intensity action was discretized into three tiers, which simplifies the true continuous dosing space, and the reward captures short-term remission gain rather than long-term outcomes such as colectomy or quality of life. Finally, the policy has not been deployed prospectively, so its causal effect remains to be established.

Future work should pursue prospective, ideally randomized, evaluation of policy-concordant dosing; richer state and action representations that include continuous herbal doses and longer-horizon rewards; multi-site external validation to characterize transportability; and integration of adherence-support interventions given the dominance of adherence among the predictors (30, 31). Coupling the policy with network-pharmacology evidence for individual herbs could further align data-driven dosing with mechanistic rationale (6, 7, 23).

## 6 Conclusion

We developed and externally validated an interpretable model for 52-week relapse in ulcerative colitis remission maintenance and a reinforcement-learning policy for titrating traditional Chinese herbal-intensity tiers. A calibrated logistic-regression model generalized best across cohorts, and medication adherence, fecal calprotectin, C-reactive protein and Coptidis Rhizoma dose emerged as the leading drivers of relapse. The net-clinical-benefit policy personalized intensification according to disease activity–sparing quiescent patients while decisively escalating those with active subclinical inflammation–and concordance between actual care and the policy was associated with a higher next-visit remission rate. By uniting transparent, well-calibrated prediction with burden-aware sequential decision-making, this work provides a reproducible framework for moving from fixed herbal regimens toward individualized, evidence-guided maintenance therapy, and it defines a concrete effect size to motivate prospective clinical evaluation.

## Data Availability

The complete, de-identified, patient-level datasets and the full analysis code supporting the findings of this study are openly available in the Zenodo repository (https://doi.org/10.5281/zenodo.20721748). The deposit provides row-level records for every participant in both the development cohort (uc_remission_cohort.csv) and the external Chengdu validation cohort (uc_external_validation_chengdu.csv), including all 21 baseline clinical, biomarker, herbal-dose and behavioral variables together with the 52-week relapse outcome, as well as the visit-level state–action–reward transitions used for the reinforcement-learning analysis (uc_rl_trajectories.csv). These are the raw, analysis-ready patient-level data rather than only aggregated or processed features; all preprocessing (median/mode imputation, one-hot encoding and within-fold standardization) is performed by the accompanying scripts, so every reported result can be reproduced directly from the raw records. No direct personal identifiers are included, consistent with the approved ethics protocol (No. 2024KL-087).
